# More Than a Pore: The Cellular Response to Cholesterol-Dependent Cytolysins

**DOI:** 10.3390/toxins5040618

**Published:** 2013-04-12

**Authors:** Sara K. B. Cassidy, Mary X. D. O’Riordan

**Affiliations:** Department of Microbiology and Immunology, University of Michigan Medical School, 1150 W. Medical Center Dr. Ann Arbor, MI 48109, USA; E-Mail: skbc@umich.edu

**Keywords:** cholesterol-dependent cytolysins, CDC, membrane repair, signaling, pore-formation, survival, cellular response

## Abstract

Targeted disruption of the plasma membrane is a ubiquitous form of attack used in all three domains of life. Many bacteria secrete pore-forming proteins during infection with broad implications for pathogenesis. The cholesterol-dependent cytolysins (CDC) are a family of pore-forming toxins expressed predominately by Gram-positive bacterial pathogens. The structure and assembly of some of these oligomeric toxins on the host membrane have been described, but how the targeted cell responds to intoxication by the CDCs is not as clearly understood. Many CDCs induce lysis of their target cell and can activate apoptotic cascades to promote cell death. However, the extent to which intoxication causes cell death is both CDC- and host cell-dependent, and at lower concentrations of toxin, survival of intoxicated host cells is well documented. Additionally, the effect of CDCs can be seen beyond the plasma membrane, and it is becoming increasingly clear that these toxins are potent regulators of signaling and immunity, beyond their role in intoxication. In this review, we discuss the cellular response to CDC intoxication with emphasis on the effects of pore formation on the host cell plasma membrane and subcellular organelles and whether subsequent cellular responses contribute to the survival of the affected cell.

## 1. Introduction

The cholesterol-dependent cytolysins (CDCs) are a family of pore-forming proteins expressed by several genera of pathogenic, primarily Gram-positive bacteria that cause very different diseases. Examples include *Streptococcus pyogenes*, a common cause of upper respiratory infections, *Bacillus anthracis*, which mostly causes cutaneous infections, and *Listeria monocytogenes*, which causes gastroenteritis. The CDCs are secreted as soluble monomers, which upon binding to host cell membranes, can oligomerize to form β-barrel pores of large diameter. Although the CDCs share structural similarity, the role of these toxins during infection largely depends on the pathogen and the context of infection. For instance, data suggest the secretion of streptolysin O (SLO) may protect the extracellular pathogen, *S. pyogenes*, from phagocytic killing by lysing target cells [1]. SLO translocates the *S. pyogenes* secreted effector, SPN, into target cells resulting in cytotoxicity [2]. In contrast, listeriolysin O (LLO) is necessary for the intracellular pathogen, *Listeria monocytogenes,* to escape the primary vacuole and replicate within the cytosol of intact macrophages [3]. Thus, bacteria can exploit a common mechanism of pore formation to drive divergent cellular outcomes during pathogenesis. 

CDC intoxication may result in some characteristic hallmarks of membrane damage, such as ion flux across the targeted cell membrane, loss of cytosolic contents, stimulation of repair mechanisms, and host cell lysis. However, many cellular responses to CDCs are poorly explained by pore-formation alone. For instance, tetanolysin O (TLO) from *Clostridium tetani,* stimulates the release of IL-1β, a pro-inflammatory cytokine, from target cells, but only at sub-lytic doses [4], which may contribute to host clearance of the bacteria. Others have shown that a non-cytolytic allele of pneumolysin (PLY) retained the capacity to stimulate IFNγ, a cytokine capable of activating macrophages, in the absence of the ability to form pores [5]. Thus, the CDCs can trigger adaptive or inflammatory pathways within the targeted cell, independently from or in addition to, the damage they cause. It is also apparent that some cell types are able to withstand and repair lesions caused by pore-forming toxins, the molecular mechanisms of which are incompletely elucidated [6]. 

Many studies have used direct mutation of the CDC protein or pre-treatment with cholesterol to assess the signal transduction capabilities of CDC treatment in the absence of pore-formation. Pre-treatment with cholesterol was reported to abrogate pore formation, but not monomer binding of LLO [7]. Typically, loss of pore-formation is measured via the lysis of red blood cells [7], the release of lactate dehydrogenase (LDH) from target cells [2,5] and/or the visualization of pores on the target cell surface [7]. However, a more recent study by Hamon and Cossart demonstrated that LLO pre-treated with cholesterol still allowed K^+^ flux across the plasma membrane, which suggests that cholesterol-treated LLO may retain some membrane damaging capacity [8]. Moreover, mutations in these pore-forming proteins that destroy their ability to lyse red blood cells may still allow some activity in other cell types, as demonstrated for the perfringolysin O (PFO) G461D mutant protein [9]. Thus, we note that caution should be taken in concluding unequivocally which host response pathways are stimulated by pore-formation and which are not, but include in our review some studies indicating that pore-formation is likely not the only property of the CDC proteins that can drive host responses.

Macromolecular pore-forming proteins are not unique to bacteria. Members of the MACPF family, including mammalian perforin and the C9 protein of the complement cascade, share strikingly similar structural topology and pore-forming mechanisms with the CDCs [10]. The parasite, *Toxoplasma gondii*, secretes a pore-forming protein, PLP1, to promote egress from infected cells [11]. *Enterolobium contortisiliquum*, a tree native to Brazil, produces a pore-forming toxin, which functions as an insecticide and deterrent from ingestion [12]. The structural similarities between CDCs, perforin, and PLP1, for example, suggest that the target cell may sense membrane damage caused by these toxins in similar ways. For instance the pores produced by both CDCs and perforin are rapidly resealed [13,14]. As such, there are likely conserved mechanisms in the adaptive cellular response to intoxication by these proteins. Given that plasma membrane disruption is ubiquitous among all forms of life, the study of the cellular response to the insult of pore formation may provide insight into broadly relevant mechanisms for cellular preservation and survival.

## 2. Physical Properties of the CDCs

### 2.1. Structure and Membrane Binding

Alignment of the amino acid sequences of CDCs reveals significant identity (from 28% to 99%) and similarity (>45%) among family members [15], suggesting conservation of activity and structure. The four CDC proteins from different bacterial species that have been crystallized in their soluble monomeric form, PFO [16], intermedilysin (ILY) [17], anthrolysin O (ALO) [18], and suilysin (SLY) [19], all display markedly similar topology (CDC membrane assembly is reviewed in detail here [20]). One of the defining characteristics of the CDC family is a structural motif of 11 residues (ECTGLAWEWWR), termed the undecapeptide or tryptophan rich motif, situated at the membrane-binding portion of the protein. Single amino acid mutations in this motif alter pore formation, but recent work suggests that other elements and not the undecapeptide are necessary for cholesterol recognition [21]. Instead, this motif is proposed to control structural reorientation after cholesterol binding [22]. 

The CDC monomers are rich in β-sheets and their structures are divided into four domains of which D2-4 are responsible for cholesterol recognition and membrane insertion. The contribution of D1 is not well characterized. For all but two CDCs, cholesterol is necessary and sufficient for membrane binding. The exceptions are ILY and vaginolysin, which require an additional receptor, human CD59, for membrane recognition [23,24], imparting species specificity to these two toxins.

### 2.2. Oligomerization and Pore Formation

Many studies support a model where CDC monomers preferentially bind in lipid rafts, which are microdomains of the plasma membrane enriched for cholesterol and signaling molecules, because of the requirement for membrane cholesterol in pore formation [25,26,27]. Coalescence of rafts containing monomers increases the likelihood of monomer-monomer interaction to promote oligomerization and subsequent pore formation. Concurrently, raft fusion is also likely to stimulate signal transduction within the cell [28]. Whether raft fusion upon monomer binding is host- or CDC-directed is unknown. It is possible that raft fusion stimulates signal transduction that occurs upon CDC membrane binding in the absence of pore formation [5], but this hypothesis has not been tested directly. It is interesting to speculate that the coalescence of lipid rafts upon CDC binding may spur signaling cascades that could function to trigger adaptive signaling mechanisms. For example, Gekara *et al.* demonstrated that treatment of cells with LLO protein deficient in pore formation, but not membrane binding, retained the ability to coalesce rafts and activate tyrosine phosphorylation [25] (some cellular responses to CDC intoxication that occur independently from full pore-formation are listed in Table 1). CDCs are not unique in using cholesterol or other components of lipid rafts for membrane binding; cholera toxin and shiga toxins use gangliosides enriched in lipid rafts as receptors [29,30]. The signal transduction stimulated by CDC binding may in part reflect a general response to raft fusion. The signaling generated upon raft fusion may then license a specific survival response, such as membrane repair in the case of CDC binding. The specificity of the response would likely be ligand-receptor and context dependent, but may reflect a universal response to membrane lateral trafficking and perturbation. 

**Table 1:** Pore-independent cellular responses to CDC treatment.

Response	Outcome	Toxin	Reference
Translocation of a bacterial effector	Hydrolysis of NAD+	SLO	[2]
Lipid raft fusion	Tyrosine phosphorylation	LLO	[25]
IFNy secretion	Inflammation	PLY	[5]
iNOS production	Anti-microbial	PLY	[5]
Histone dephosphorylation	Transcriptional modification	LLO	[71]
Vaccine adjuvant	Improved tumor clearance	LLO	[97]
MHC presentation	Adaptive immunity	LLO	[96]

When CDC monomers interact in the membrane, they undergo extensive changes in their secondary structure for proper membrane insertion orientation [31,32]. As monomers oligomerize they form a ring on the cell surface, termed the pre-pore complex. Pore formation occurs upon a conformational change that drives insertion of a β-barrel hairpin into the membrane bilayer. [33]. Much of our understanding of the physical characteristics of the CDC pores comes from cryo-electron microscopy and atomic force microscopy visualizing toxin pores on membranes [34,35,36], and these micrographs reveal both fully formed circular pores and what appear to be partial pores, or arcs. The argument could be made that the arcs arise as artifacts of sample preparation and non-native membrane conditions. However, there is evidence to suggest that some CDCs may form arcs under native conditions as well. A study by Shaughnessey *et al.* demonstrated the sequential release of a small dye (Lucifer Yellow, *MW* 522), termed perforation, followed by a large dye (TRDx average *MW* 10,000) in *Listeria*-containing phagosomes of macrophages. This perforation of the phagosomal membrane required the *L. monocytogenes* CDC, LLO [37]. These data suggest that lesions of different sizes occur in the phagosomal membrane during infection. It is possible that CDC arcs in the membrane allow the leakage of the smaller dye, whereas full pores, which would have a greater diameter, allow for loss of the larger dye. Others have provided evidence supporting the biological existence of CDC arcs as well [34,38,39]. Using genetic techniques, Palmer *et al.* demonstrated that SLO mutant proteins that preferentially formed arcs prevented the influx of large dextran molecules into erythrocytes, whereas SLO proteins that preferentially formed circular pores did not [38]. The role of arcs in the cellular response to toxin is not well understood. Nonetheless, the formation of arcs might trigger different signaling mechanisms and cellular responses compared to formation of the full pore, which forms a channel lined with protein. The uneven disruption of the plasma membrane during arc insertion would expose hydrophobic sections of membrane to the cytosol. This hydrophobicity could then serve as a danger signal to activate an immune response [40], a repair response, or both. 

The full CDC pore is large, composed of ~35–50 monomers with a diameter of 250–300 Å, and has been used as a delivery mechanism to introduce foreign matter, such as antibodies, into the cytosol [41]. Despite their size, the holes formed by CDCs are rapidly sealed [13,42], suggesting that the host cell has efficient adaptive mechanisms to withstand this type of membrane attack. 

## 3. Membrane Response to CDC Intoxication

### 3.1. Endocytosis of Pores

**Figure 1:**
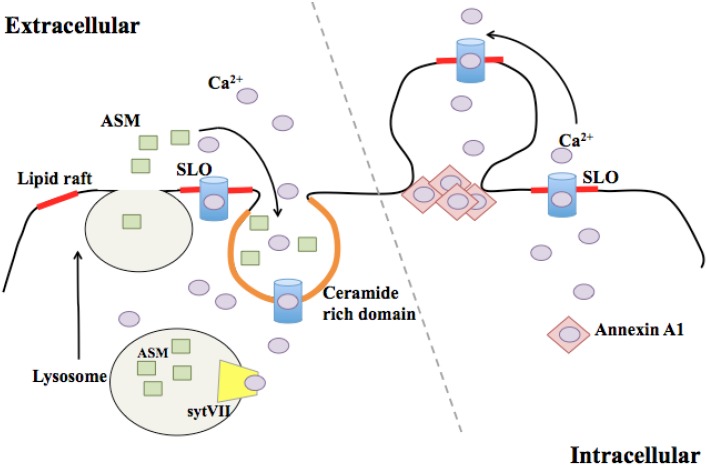
CDC pore-dependent membrane repair. A schematic of endocytosis and ectocytosis in response to CDC-induced plasma membrane damage. The Streptolysin O (SLO) pore (blue barrel) allows for the influx of Ca^2+^ (light purple) into the cytosol. Synaptotagmin VII (sytVII, yellow) binding of Ca^2+^ stimulates the fusion of lysosomes with the plasma membrane. Fused lysosomes release acid sphingomyelinase (ASM, green) into the extracellular space. ASM converts sphingomyelin in the membrane to ceramide, which allows for invagination and endocytosis of toxin into the cell. Damaged caused by the SLO pore can also stimulate ectocytosis. Increases in cysolic Ca^2+^ are sensed by annexin A1 which aggregate at the neck of the blebbed membrane to limit cytosolic leakage from the damage bilayer.

Once a CDC pore is inserted into the membrane, the damaged cell must heal or remove the lesion to maintain viability. One mechanism to achieve this is to internalize the damaged section of membrane via endocytosis (Figure 1). Work from Norma Andrews and colleagues has demonstrated that when SLO inserts into the membrane, ion flux across the damaged membrane stimulates lysosomal exocytosis, delivering additional membrane to the vicinity of the lesion [43]. This process is critically dependent on Ca^2+^ [44], as is the resealing process [13]. Lysosomes contain acid sphingomyelinase (ASM), which hydrolyzes sphingomyelin to form ceramide. Release of ASM into the extracellular space after lysosomal fusion with the plasma membrane generates a ceramide-rich platform that can invaginate to initiate endocytosis [45,46]. However, the molecular mechanism that pinches off invaginated membrane for endocytosis is not fully understood. Sphingomyelin tends to cluster with cholesterol in the plasma membrane [47], and thus has a high likelihood of being in close proximity to cholesterol-bound CDC pores. The ceramide-rich domains generated by ASM release can spatially reorganize receptors to activate intracellular signaling [48]. Therefore, the generation of ceramide-rich platforms may also act as a mechanism by which the cell can signal during CDC intoxication. Once internalized, SLO-containing vesicles traffic through the endocytic pathway to promote the degradation of the pore-forming protein [49]. 

This model of repair is not unique to CDC-mediated membrane damage, as introducing a scratch wound on the cell also stimulates lysosome fusion and rapid repair [50]. However, this model does not apply to all types of PFT damage. For instance, pores formed by aerolysin, a small heptameric toxin whose pore size is 1.5–2 nm, from *Aeromonas hydrophila*, are very stable in the membrane [51], and cause prolonged intracellular ion dysregulation compared to CDCs [42]. 

### 3.2. Membrane Blebbing

Membrane blebbing, or ectocytosis, is commonly used as a hallmark of apoptosis [52], but blebbing in viable cells can also promote motility and cytokinesis [53]. Blebbing occurs when the membrane separates from the cytoskeletal scaffold, which confers rigidity to the bilayer when attached. Cytoplasmic hydrostatic pressure causes the outward protrusion of detached membrane to form a spherical bleb (Figure 1). Recent data suggest that membrane blebbing can also protect cells from CDC-induced lysis. Babyichuk *et al.* demonstrated that treatment of cells with SLO induced membrane blebbing, and that this process, much like lysosomal exocytosis, was dependent on sufficient Ca^2+^ concentration in the extracellular medium [54]. Blebbing protected intoxicated cells by isolating the damaged section of membrane, and cells that underwent blebbing were more likely to survive the insult [54]. These experiments supported blebbing as a process by which cells could protect themselves from membrane perturbation. Keyel *et al.* used “deep-etch” electron microscopy to demonstrate that blebs formed in response to SLO bud from the cell surface [55]. The shed vesicles appeared to be enriched for SLO protein compared to lysates derived from whole cells. The authors concluded that membrane blebbing promotes the shedding of SLO-damaged membranes to promote cell survival [55]. Therefore, the cellular response to CDC intoxication, in this case membrane blebbing and shedding, can promote the survival of the affected cell. The host pathways that control detachment of the plasma membrane from the cortical cytoskeleton to promote blebbing during intoxication have yet to be determined. Additionally, the mechanisms by which cells that undergo blebbing and shedding replenish the plasma membrane are not known. Recent data revealed a role for caspase-7 in promoting plasma membrane integrity during CDC intoxication [56]. Since caspases promote blebbing during apoptosis [57,58], it is possible that CDC-induced and apoptosis-associated blebbing are governed by a shared molecular mechanism, although this has yet to be tested directly. 

The two mechanisms of membrane repair outlined here, endocytosis and ectocytosis, are not inherently discordant. Although Keyel *et al.* found no evidence of endocytosis in their experiments [55], it is possible that different cell types favor one mechanism over the other, or that the amount of damage dictates the predominant repair mechanism. It is also possible that these two mechanisms work in concert. Cells that bleb and shed damaged membrane would likely need to replace that membrane to maintain cell surface area and hydrostatic homeostasis, and lysosomal exocytosis could supply this membrane faster than making new membrane *de novo*. Additionally, in all cases, extracellular Ca^2+^ was critical for repair. In the endocytosis model, synaptotagmin VII, a calcium binding protein present in lysosomes, was required for directing the fusion of these vesicles with the plasma membrane for lesion repair [50]. In the ectocytosis model, annexins, a family of calcium binding proteins present in the cytosol, act as a plug at the neck of the bleb to seal off the contents of the cytosol away from the damaged membrane [54]. Different concentrations of intracellular Ca^2+^ may trigger different mechanisms of membrane repair, or the relative abundance of calcium sensors in different cell types may drive one repair response over another. 

## 4. Intracellular Effects of CDC Intoxication

### 4.1. Effects on the Endoplasmic Reticulum and Golgi

Beyond the effects of CDC perturbation at the plasma membrane, there are hints that CDCs can also affect intracellular compartments. Pillich *et al.* recently published a report suggesting that infection with *L. monocytogenes* can activate the unfolded protein response (UPR) of the endoplasmic reticulum (ER) [59]. The UPR is a stress response which acts to balance protein synthesis and protein folding in the ER to maintain cellular homeostasis [60]. UPR activation was dependent on secretion of LLO by *L. monocytogenes* [59]. Others have shown that UPR activation during intoxication with other pore-forming toxins is protective at the level of the organism. Loss of *ire-1* or *atf-6*, two UPR sensors, in *Caenorhabditis elegans* results in hypersensitivity to the PFT, Cry5B [61]. How activation of the UPR protects cells during intoxication is not known, but there are several possible explanations. UPR stimulation activates autophagy [62], a degradative pathway responsible for the turnover of cellular contents and organelles, and autophagy may contribute to the degradation of PFTs, damaged membrane, or both. UPR activation can also stimulate lipid synthesis [63], which could enhance membrane repair and replenishment after toxin-induced damage. Finally, the UPR promotes increased vesicular traffic between the ER and the Golgi [64]. Vesicles derived from these compartments may contribute to membrane repair in a manner analogous to lysosomal exocytosis. 

**Figure 2:**
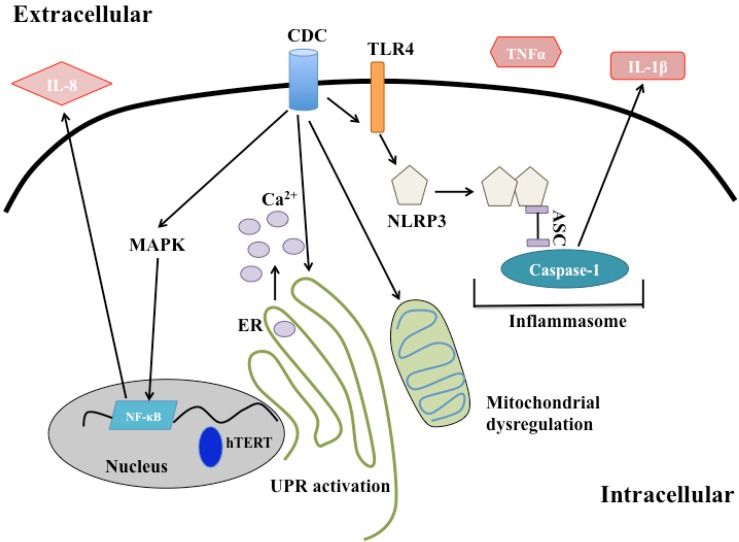
CDC pore-dependent signaling responses. A schematic of the different signaling responses activated by CDC damage. CDC intoxicated cells activate the inflammasome, stimulating the release of IL-1β. CDC perforated cells also secrete TNFα. Calcium is released by the ER and the UPR is activated in response to intoxication. CDC treatment results in loss of mitrochondrial membrane potential and transient fragmentation of the mitochondrial network. IL-8 is secreted in a MAPK and NF-κB dependent manner.

In addition to activation of the UPR, there are some clues that suggest CDCs act on the ER membrane directly. PFO perforates the ER [65] despite the low concentration of cholesterol in this bilayer [66]. LLO stimulates the rapid release of Ca^2+^ from the ER of mast cells by Ca^2+^ channel-dependent and -independent mechanisms [67] (Figure 2), indicating that LLO may act on the ER membrane as well. However, LLO pores were not directly detected on the ER membrane [67]. Although CDC binding to internal stores of membrane is an appealing model to explain aspects of CDC-mediated signaling, most of the data remains speculative. It is inherently difficult to tease apart the cellular response to CDC-damaged organelles from the signaling that occurs as a result of plasma membrane damage, since the former cannot be detected without inducing the latter. Better tools are needed to determine if CDCs act on intracellular compartments directly, and whether these organelles can drive CDC-specific responses in the absence of plasma membrane damage. 

Although the Golgi and ER are intimately connected within the cell, less is known about CDC interactions with the Golgi. Treatment of cells with SLO increases Golgi-derived trafficking to the damaged membrane [68], but this response is not likely specific to CDC-intoxication. Nonetheless, given the critical role for the Golgi apparatus in lipid metabolism and transport [69], the Golgi may function to modulate responses to CDC-derived damage. Infection with some bacterial pathogens has shed light on Golgi-plasma membrane interactions. Divangahi *et al.* elegantly demonstrated that *Mycobacterium tuberculosis* infection induces plasma membrane damage and requires translocation of Golgi-derived vesicles to the bilayer to successfully seal lesions [70]. Thus, vesicular traffic from the Golgi represents an attractive source of new membrane and newly synthesized lipids to replace membrane lost during endo- or ectocytosis in response to CDC perturbation. 

### 4.2. Effects on the Mitochondria and Nucleus

CDCs exert effects on many intracellular compartments. For instance, Goldmann *et al.* determined that SLO treatment of macrophages had profound effects on the mitochondria of the cell, including loss of mitochondrial membrane potential, which led to the oncotic death of the intoxicated cell [71]. Interestingly, loss of plasma membrane integrity was not the sole trigger for mitochondrial dysfunction, as cell death was not inhibited by increased osmolarity in the culture media. Also, the pore size at the plasma membrane was significantly smaller than what is estimated for the SLO pore [71], indicating that either the pores were of mammalian origin, or that SLO arcs might be the predominant form of damage. Together, these results suggest that SLO potentially acts as a signaling molecule, in addition to its role as a PFT, with negative implications for the function of cellular mitochondria. 

Another study showed that PLY intoxication affects the mitochondria of neuronal cells to induce apoptotic cell death, and PLY protein was detected on the mitochondrial membrane using immuno-EM [72]. The authors demonstrated that the ability to form pores at the plasma membrane was necessary to induce mitochondrial dysregulation, but whether PLY protein formed pores on the mitochondrial membrane was not assessed. It is possible that monomer binding to the mitochondrial membrane was sufficient to induce the response, and that pore-formation at the plasma membrane was needed to introduce monomers into the cytoplasmic space. Considering that the sterol content of the mitochondrial membrane is very low [66], it is plausible that PLY oligomerization in this membrane would also be low. 

LLO alters the mitochondria as well, but differently from SLO and PLY. Stavru *et al.* demonstrated that treatment of cells with non-lytic concentrations of LLO induced fragmentation of the mitochondrial network [73] (Figure 2). Although mitochondrial fragmentation is sometimes used as a hallmark of programmed cell death, markers of apoptosis were not observed in these cells. In fact, the LLO-induced changes in mitochondrial morphology were transient, leading the authors to hypothesize that altering mitochondrial dynamics would slow the metabolism of the cell to promote the early stages of *L. monocytogenes* infection [73]. Whether other CDCs induce a similar morphological change is not known. The authors implicated Ca^2+^ flux as important in eliciting this response [73]. In conclusion, it is clear that intoxication with CDCs can significantly alter the mitochondria of intoxicated cells, but the outcome of that alteration is likely cell type- and potentially CDC-specific. 

Most of the existing knowledge of the effects of CDCs on the nucleus has come from the exogenous treatment of cells with LLO. LLO binding to the plasma membrane can stimulate specific dephosphorylation of histone H3 in the nucleus [74]. PFO and PLY were also able to spur histone modification, but treatment with detergents and an unrelated PFT was not, indicating that the response is specific to CDC membrane binding. These data supply direct evidence for the hypothesis that CDCs can act as signal transducers. Using microarrays to monitor changes in transcription, Hamon *et al.* found that epigenetic changes caused by LLO binding resulted in altered expression of ~150 genes [74]. The authors found that pre-treatment of LLO with cholesterol inhibited the ability of the CDC to stimulate the release of LDH from these cells, but not the dephosphorylation of histone H3, indicating that full pore formation was not needed to induce this response. In a subsequent paper Hamon *et al.* determined that cholesterol-treated LLO retained the ability to stimulate K^+^ flux across the plasma membrane, and that this ion flux was sufficient to stimulate histone modification [8]. 

Treatment of cells with LLO can induce the nuclear translocation of the master transcriptional regulator, NF-κB [75], as well as activate the MAPK cascade [76], both of which have the capacity to globally alter gene transcription and activate immune signaling (discussed below). Recent data suggest that LLO stimulates degradation of human telomerase reverse transcriptase [77], although that regulation is due in part to Ca^2+^ flux as a result of membrane perturbation. Taken together, these data suggest that CDCs can have profound influence on transcriptional regulation of the intoxicated cell, which would likely affect the outcome of infection. 

### 4.3. Endolysosomal Network

The endolysosomal network is a key cellular defense system that traps bacterial pathogens within a membrane bound compartment through phagocytosis or autophagy, and a CDC toxin, LLO from *L. monocytogenes*, is crucial for escape from this restrictive environment [78]. LLO perforates the phagosome, stimulating ion flux and cellular signaling [37,79]. One outcome of LLO perforation of the phagosome is to delay phagosome maturation [80]. While escape is the primary function of LLO-induced membrane damage within the vacuole, it is conceivable that pore formation permits the passage of other *L. monocytogenes* virulence factors, like PI-PLC and PC-PLC, across the phagosomal membrane to trigger host signaling pathways [81,82]. Phagosomal membrane damage induced by LLO activates autophagy, a scavenging pathway that targets cytoplasmic components for lysosomal degradation [83,84]. Although LLO is the only CDC expressed by a pathogen that is largely intracellular, some extracellular pathogens expressing CDC toxins, like *S. pyogenes*, can be found within host cells and their entry and survival may be influenced by CDC expression [85,86]. The structurally related mammalian MACPF protein, perforin, which confers cytolytic activity to T lymphocytes and natural killer cells, also acts from within the endolysosome, delivering granzyme B to the cytosol of infected target cells [87]. Damage to the lysosomal compartment, as a consequence of pore formation, is a potent trigger of the inflammasome resulting in secretion of the pro-inflammatory cytokine IL-1β [88,89]. Thus, LLO pore formation is critical for *L. monocytogenes* to escape the endosomal compartment, but in doing so alerts the host to its presence.

### 4.4. Immune Signaling

There is a large body of evidence that cells can mount an inflammatory response to non-lytic intoxication with CDCs, and that CDCs themselves may act as pathogen-associated molecular patterns (PAMPs). Intoxication with LLO can activate the MAPK pathway [76], a signaling cascade that promotes both innate and adaptive immune responses [90]. Nasopharyngeal epithelial cells secrete a neutrophil chemoattractant, IL-8, upon intoxication with PLY, which is dependent on MAPK signaling [91]. SLO intoxication of neutrophils stimulates their secretion of antimicrobial peptides and inflammatory proteins via the activation of the MAPK cascade [92]. Treatment of macrophages with heat killed bacteria and either LLO or SLO activates MAPK-dependent secretion of IL-10 [93], an anti-inflammatory cytokine, which suggests that the immune response to CDC intoxication is tempered by the presence of additional stimuli.

In the early 1990s, it was demonstrated that monocytes secrete TNFα and IL1β in response to treatment with SLO or PLY [94,95]. Approximately 10 years later, this cytokine release was linked to Toll-like receptor signaling, as macrophages lacking Toll-like receptor 4 (TLR4) failed to respond to purified PLY [96]. Similar results were obtained treating macrophages with recombinant ALO, LLO, PFO, and SLO [97]. Blocking the binding of lipopolysaccharide to TLR4 did not abrogate cytokine secretion, suggesting that activation of TLR4 by these toxins was not due to endotoxin contamination [97]. Whether CDCs act directly as a ligand of TLR4 is still not known. Another possibility is that binding or oligomerization of CDCs at the plasma membrane induces rearrangement of membrane microdomains. Coalescence of lipid microdomains could promote TLR4 dimerization spurring immune signaling [98]. In this way, binding or oligomerization of CDCs at the membrane may enhance PAMP-like signaling cascades. 

In addition to activation of pattern recognition receptors at the cell surface, CDCs can also stimulate intracellular immune signaling. TLO treatment of LPS-primed macrophages activates the NLRP3 inflammasome, inducing the processing and secretion of IL-1β [4]. The authors concluded that full pore formation was necessary for cytokine secretion as pretreatment of the toxin with cholesterol was insufficient to stimulate the response. Whether cholesterol-treated TLO retains the ability to alter intracellular K^+^ similar to cholesterol-treated LLO [8] has not been studied. Later it was determined that SLO-induced inflammasome activation required TLR signaling, but infection with the bacterial pathogen that produces the toxin, *S. pyogenes*, does not [99]. Therefore, TLR signaling may represent a mechanism by which macrophages can distinguish between intoxication and direct infection with a particular pathogen in order to guide the proper immune response. It is important to note that NLRP3 can be activated by low intracellular K^+^ [100] and treatment of cells with CDCs can result in the depletion of cytosolic K^+^ [41]. Thus, activation of NLRP3 could represent a general response to membrane damage, which could be altered by additional virulence factors secreted by the bacterium or by a dynamic host response in the context of infection. However, murine splenic cells secrete IFNγ and produce iNOS in response to mutant PLY that appears to lack the ability to form pores [5], so cytokine secretion in response to intoxication with CDCs may have damage-dependent and damage-independent triggers. 

Treatment of immune cells with CDCs can modulate adaptive immune function. When macrophages are exposed to SLO-rich plasma membrane-derived vesicles, they secrete less TNF upon LPS stimulation and present fewer peptides to B cells [101]. Non-hemolytic LLO has potent immunogenic [102] and adjuvant properties for anti-cancer vaccination [103]. LLO-derived epitopes were presented to CD4^+^ and to a lesser extent CD8^+^ T cells [102], an interesting finding since CD8^+^ cells are responsible for anti-tumor immunity as well as *L. monocytogenes* clearance [103,104,105]. However, when CD4^+^ T cells were directly exposed to LLO that retained pore-forming capabilities, these cells became anergic [106] or apoptotic [107]. Therefore, the relative levels and activity of CDC toxin present during infection, as well as its cellular context, regulate the magnitude and outcome of the resulting immune response.

## 5. Conclusions

An intact eukaryotic cell membrane is critical for cell survival and yet this barrier is routinely assaulted by CDC toxins during infection. Studies using these toxins have elucidated previously uncharacterized membrane repair mechanisms that also function to repair mechanical stresses, such as scratch wounds. Therefore, the damage responses discovered as a result of CDC pore-formation may shed light on the mechanisms cells use to protect the bilayer in response to diverse insults. Studies on the morphological and transcriptional differences that occur during CDC treatment of cells will continue to guide our understanding of the host response to intoxication. Finally, signaling cascades activated by target cells upon CDC exposure indicate these toxins may be important modulators of innate and adaptive immunity, in addition to their specific functions in virulence. The CDC toxins have been a valuable model to study key aspects of host-pathogen interactions and continue to be versatile tools to investigate the cellular response to damage and infection, improving our understanding of host-pathogen interactions and immunity.
